# Fever of Unknown Origin: An Unusual Case

**DOI:** 10.1155/2011/271808

**Published:** 2011-06-30

**Authors:** R. A. Bansal, G. R. Hayman, A. S. Bansal

**Affiliations:** Department of Immunology, Epsom and St Helier University Hospitals NHS Trust, Carshalton, Surrey SM5 1AA, UK

## Abstract

Recurrent episodic fever of unknown origin (FUO) arising from tumour of the gastrointestinal tract is rare. We report an otherwise healthy 62-year-old man with recurrent circumscribed bouts of fever and raised CRP for 3 years who has remained well and fever-free 2 years after the removal of a well-differentiated adenocarcinoma of the colon. Occult colonic neoplasm should be considered and sought when routine investigations for FUO are negative.

## 1. Case Report

A 62-year-old man presented with a 3-year history of recurrent episodes of high fever up to 40°C accompanied by rigors, sweating, flu-like sensation, and malaise. These episodes occurred roughly 3-4 times a year and would last between 2 to 3 days. A markedly elevated C-reactive protein of up to 64 mg/L but without a leucocytosis or elevation of the ESR was evident with the most closely monitored of the episodes. Risk factors for brucellosis, endocarditis, tuberculosis, travel to tropical countries, and retrovirus infection were negative. The patient denied anorexia and weight loss, and there was no family history of fevers, lymphoproliferative disease, or cancer. A complete physical examination was normal and without lymphadenopathy, heart murmurs, clubbing of the nails, skin rashes, joint effusions, and synovitis. 

A CT scan of the chest and abdomen was completely normal as was a barium enema performed 2 years earlier. Urine cultures were negative for growth. Numerous blood tests were returned as normal or negative. These included full blood count and blood film analysis, several blood cultures, creatine kinase, and lymphocyte subset analysis, as well as tests of organ-specific autoimmunity on a liver, kidney, stomach tissue block. There was also no evidence of antinuclear antibodies, rheumatoid factor, and antineutrophil cytoplasmic antibodies. Serum immunoglobulins were normal, and there was no abnormality on serum immunoelectrophoresis. The kappa and lambda free light chain estimation by Freelite (Binding Site, UK), serum-angiotensin-converting enzyme, quantiFERON for mycobacterial infection and tests of liver and renal function as well as lactate dehydrogenase were also either normal or negative. 

Following a routine positive faecal occult blood analysis, the patient underwent colonoscopy and an adenocarcinoma of the sigmoid colon was discovered. This was recorded as a stage 1 sigmoid adenocarcinoma without lymph node involvement and without abscess formation or necrosis (T1N0M0, Dukes A). The patient made an uneventful recovery and has remained free of further bouts of fever for the previous 2 years.

## 2. Discussion

Pyrexia or fever of unknown origin (FUO) is often defined as fever of more than 38.3°C for 3-week duration and without an identifiable cause despite intensive investigations for more than one week. While fever can frequently be traced to infection, systemic autoimmunity and inflammatory disease, malignancy involving the lymphoreticular system is not an unusual cause [1] and solid organ tumours such as those of the kidneys and liver are not infrequently implicated. However, other solid organ malignancies are rarely causative of fever unless abscess formation supervenes. This was considered aetiologically important in the three patients with FUO and colonic carcinoma reported by Agmon-Levin et al. [2]. In each case a microcytic anemia was accompanied by histological evidence of “a severe organized inflammatory process forming abscesses in the pericolic fat.” In the case reported by Karachalios et al. [3] abscess formation was absent but the fever and the highly elevated ESR that accompanied the rectosigmoid carcinoma was evident continuously for two months. Importantly, however, these abnormalities, as well as the patient's fatigue and sweats, disappeared completely after a left sigmoid colectomy and during eight years of followup. A similar pattern was also observed in the patient reported by Ilan and Shalit [4], whose six months of FUO disappeared after a well-differentiated adenocarcinoma of the sigmoid colon was removed. 

In contrast to the aforementioned cases, the present patient had suffered recurrent circumscribed episodes of acute high fever lasting 2 to 3 days each and with elevated parameters of inflammation for two years. This was also observed in the 4 cases of colonic carcinoma reported by Fernández Guerrero et al. in 2002 [5] and attributed to intermittent E. coli bacteraemia. However, the problem is clearly rare as it was recorded as only a single case in the 110 cases of FUO reported by Barbado Hernández et al. [6]. Further 2 cases have been reported as a brief report by Rivero Marcotegui et al. [7] and a single case by Sánchez Rodríguez et al. [8]. In each of these cases, patients without GI symptoms had suffered recurrent bouts of fever lasting 1–3 days. The bouts had occurred every 10–20 days and the subsequent removal of a well-differentiated adenocarcinoma of the colon led to a cessation of the bouts of fever despite long followup. In one of these cases reactive lymphadenitis was found at operation and the role of interleukin- (IL-) 1 was discussed [8]. 

The mechanism of the intermittent fever and raised parameters of inflammation in patients with a well-differentiated solid tumour is unclear. However, cytokines such as IL1, IL6, IL18, and tumour necrosis factor *α* are very likely involved as indeed is prostaglandin E2 [9]. It is possible that specific polymorphisms of the promoter regions of these proinflammatory cytokines or other pathways involved in the generation of fever may predispose certain patients to this problem. The factor(s) that stimulates the synthesis and secretion of these cytokines remains unclear although intermittent endotoxaemia or bacteraemia remain possible. Why subjects of Spanish origin constitute the majority of patients detailed in the previous reports of this condition is unclear.

## 3. Conclusion

Tumours of the gastrointestinal tract should be considered a cause for FUO when routine imaging, blood/bone marrow analysis, tests of systemic autoimmunity and microbial cultures are normal or negative. In this event testing for faecal occult blood and colonoscopy should be considered and even in the absence of gastrointestinal symptoms and iron deficiency.

## 4. Learning Points

Infection, systemic autoimmunity, inflammatory disease, malignancy involving the lymphoreticular system, and tumours of the liver and kidney are common causes of FUO. On rare occasions tumours of the gastrointestinal tract can also cause fever and often in a sporadic fashion. An early faecal analysis for occult blood should be undertaken if the cause of an FUO remains elusive after conventional investigation.
